# School victimization and Internet addiction among Chinese adolescents: The mediating roles of life satisfaction and loneliness

**DOI:** 10.3389/fpsyg.2022.1059486

**Published:** 2023-01-12

**Authors:** Xinxin Shi, Rulin Wang

**Affiliations:** ^1^School of Education, Zhengzhou University, Zhengzhou, China; ^2^School of Marxism, Zhengzhou University, Zhengzhou, China; ^3^Faculty of Psychology, Beijing Normal University, Beijing, China

**Keywords:** school victimization, life satisfaction, loneliness, Internet addiction, adolescents

## Abstract

The present study investigated the possibility of life satisfaction and loneliness mediating the link between school victimization and Internet addiction. A total of 3,363 middle/high school students (45% males; *M_age_* = 15.67 years old, *SD* = 1.58) completed a series of self-report questionnaires, which included school victimization, life satisfaction, loneliness, and Internet addiction. The findings demonstrated a positive relationship between school victimization and Internet addiction. In addition, life satisfaction and loneliness mediated the link between school victimization and Internet addiction. Overall, these findings contribute to a better understanding of the association between school victimization and Internet addiction. They also extended the GST, providing suggestions for preventing and managing adolescents’ Internet addiction.

## Introduction

School victimization is a long-standing and thorny issue. Studies from different countries have described the prevalence of victimization (Wolke et al., 2001; Delfabbro et al., 2010; Sánchez-Queija et al., 2017). A cross-national survey of students from 40 nations also found that 12.6% reported they were school bullying victims (Craig et al., 2009). According to Chinese national research, 10.89% of adolescents were victims (Luo et al., 2022). These findings demonstrate that school victimization is a global problem that has proved challenging to deal with during the previous three decades.

School victimization has a wide range of detrimental developmental consequences. Victims reported high levels of anxiety and depression (Stapinski et al., 2015), loneliness (Carney et al., 2020), poor psychological adjustment (You and Bellmore, 2012), lower levels of happiness and life satisfaction (Estévez et al., 2009; Aunampai et al., 2022), lower levels of self-esteem (Overbeek et al., 2010), and even suicidal risks (Xiao et al., 2022). Mainly, victims frequently exhibit other problematic behaviors, which attract greater attention from instructors or parents and cause them to ignore the causes of these issues. This attention bias will fail to change the victim’s problematic habits and increase the likelihood of victimization again due to misunderstanding.

The Internet provides victims with solace by giving them anonymity and a sense of detachment from reality. However, it may also lead to addiction and harm their health (Hossin et al., 2022). The general strain theory (Agnew, 2001) states that school victimization will lead to victims having a negative opinion of themselves, their peers, and the school, then negative emotions, and eventually cause the development of delinquent behaviors, such as dedicating their time to the Internet. Some studies supported the link between school victimization and Internet addiction (Guo et al., 2020), but further research is required to determine the exact process.

### School victimization

Olweus (1996) defined school victimization as “a student is being bullied or victimized when he or she is exposed, repeatedly and over time, to negative actions on the part of one or more other students.” Although the precise definition of school victimization is still debatable, researchers have agreed on some traits (Goldsmida and Howie, 2014): (1) repetition (Baldry and Farrington, 2004); (2) victimization distress; (3) intention to harm (Anderson and Bushman, 2002); (4) power inequity (Salmivalli and Nieminen, 2002). In addition to physical victimization, school victimization also includes verbal and psychological violence, such as humiliation, isolation, rumor, and name-calling, which most frequently occur in schools and are always ignored (Zhang et al., 2019).

Based on the definition, it is reasonable to infer that victims are always frightened to seek help and suffer from various issues, which has been validated in earlier work (Kaltiala-Heino et al., 2000; Luk et al., 2010). However, the specific effect on victims varies. Some victims experience internalizing symptoms (e.g., sadness, anxiety, and loneliness; Reijntjes et al., 2010), while others act out and have externalizing issues. For example, they may attack people or engage in something to vent their feelings or relieve pain (Reijntjes et al., 2011; Richard et al., 2020).

### School victimization and Internet addiction

The Internet is a crucial part of our civilization in this age of information and technology. Excessive Internet use, on the other hand, can lead to Internet addiction, which can be hazardous to one’s physical or mental health (Tsai and Lin, 2003). Internet addiction is also a severe problem among adolescents (Chi et al., 2020). Thus, researchers conducted studies and intervention programs to reduce adolescents’ Internet addiction. They discovered that some family and school factors might cause adolescents’ Internet addiction (Wang et al., 2017), such as impaired family functioning (Shi et al., 2017), negative parenting styles (Li et al., 2018), poorer teacher-student relationships (Jia et al., 2017), and negative peer relationships.

Agnew’s general strain theory (GST) was first proposed to explain delinquency, which was also used to explain adolescents’ Internet addiction. It suggests that adolescents are obliged to stay in specific situations (for example, family and school) and strain due to the blocking of pain-avoidance behavior (Agnew, 1985, 2001). When exposed to strain, adolescents strive to avoid painful or aversive events that may lead to illegal escape efforts or anger-based misbehavior, such as excessive drug use or problematic Internet use. A recent study extended GST to Internet addiction and revealed that academic stress might enhance the chance of adolescents getting Internet addiction (Jun and Choi, 2015). School victimization is also a type of strain, so GST may be used to describe the effect of school victimization on Internet addiction. Thus, GST may be the theoretical basis to explain the association between school victimization and Internet addiction.

Some studies also supported the direct and positive link between school victimization and Internet addiction (Guo et al., 2020). A longitudinal study indicated that cyberbullying victimization among Spanish adolescents at T1 positively predicted problematic Internet use at T2 (Gámez-Guadix et al., 2013). Recently, Zhai et al. (2019) investigated adolescent students in China and confirmed that victimization experience influenced the development of problematic Internet use. Based on theoretical and empirical evidence, we assume that school bullying frustrates and imprisons the victims. The Internet is offered as a method to manage negative emotions and escape from unpleasant reality, resulting in an addiction to the Internet. Thus, we hypothesize that school victimization will be associated with Internet addiction positively (H1).

### The mediating role of loneliness

GST indicated that strain did not lead to delinquency directly. It would elicit negative emotions first, then cause misbehavior, such as problematic Internet use. Loneliness is a common negative emotion that victims frequently experience. It results from being cut off from social networks and being perceived as unpopular among peers (Newman et al., 2005). Some researchers pointed out that school victimization caused loneliness, whereas other scholars stated that bullies are more likely to target unpopular and isolated peers. However, studies confirmed the significant positive association between school victimization and loneliness (Pengpida and Peltzerb, 2019). Kochenderfer-Ladd and Wardrop (2001) conducted a four-year longitudinal study with 388 students and identified that victims were initially depressed but might still have friends. However, as time passes, both avoidance by peers and adversity that few people help would remind them that peers might not like them. Hence, victims gradually developed a sense of loneliness. Other studies have found that victims experienced more loneliness than non-victims (Li et al., 2019).

Loneliness is also a strong predictor of Internet addiction (Yao and Zhong, 2014). Western studies have shown a positive association between Internet use and loneliness, in which people with higher levels of loneliness will have excessive Internet use (Esen et al., 2013). Zhang et al. (2018) conducted a longitudinal analysis in China and discovered that loneliness significantly impacted Internet addiction. Taken together, school victimization, combined with loneliness, may increase the likelihood of becoming an Internet addict. Therefore, we hypothesize that loneliness may mediate the association between school victimization and Internet addiction (H2).

### The mediating role of life satisfaction

Life satisfaction is defined as a person consciously evaluating one’s life aspects (Pavot and Diener, 1993). There are numerous dimensions of life satisfaction, but school, self, and friend satisfaction are more prominent for adolescent students.

School is a situation where adolescents spend the majority of their time. School victimization will undoubtedly impact their school experience and lead to alienation from friends. Varela et al. (2016) used a sample of 802 students to demonstrate that adolescents exposed to school victimization had lower school satisfaction. Furthermore, Kerr et al. (2011) demonstrated that friend satisfaction and self satisfaction are negatively associated with school victimization. According to Kaltiala-Heino et al. (2000), victims will fail to receive support from social networks, which may cause isolation from peers and thus reduce life satisfaction. Another possibility is that school victimization causes adverse mental disorders, which decreases perceived life satisfaction (Yang et al., 2021).

In addition, Internet use is considered a form of self-medication (e.g., it can reduce one’s negative moods; Senol-Durak and Durak, 2010). A higher level of life satisfaction represents individuals’ positive emotional responses (Sung-Mook and Giannakopoulos, 1994). As a result, life satisfaction precedes Internet addiction (Longstreet and Brooks, 2017). Kabasakal (2015) verified that lower life satisfaction increased the likelihood of problematic Internet use. Based on the findings thus far, we hypothesize that life satisfaction may mediate the relationship between school victimization and Internet addiction (H3).

### The relationship between life satisfaction and loneliness

Previous studies have confirmed the negative correlations between life satisfaction and loneliness (Şahin, 2013). Since cognition and emotion are inseparable, thus the present study not only considers the mediating roles of life satisfaction and loneliness, respectively but also aims to examine the chain mediation effect between the two variables.

The cognitive theory of emotions asserts that cognitive evaluation influences emotion (Lazarus, 1991). Heinrich and Gullone (2006) also supposed that loneliness is an emotionally unpleasant experience with a cognitive component. First, qualitative or subjective appraisals of social relationships will affect loneliness (Asher and Paquette, 2003). Belongingness is an essential need for humans, so loneliness may result from having a weaker sense of belonging. Life satisfaction is a subjective assessment of one’s quality of life, such as being unsatisfied with oneself, peers, and school will lead to poorer interpersonal relationships and cause a high level of loneliness. Second, according to attribution theory, the irrational cognitive style and attribution style (usually uncontrollable, internal, and stable attribution) may result in loneliness when faced with the discrepancy between expectation and reality (Vanhalst et al., 2015). People with low life satisfaction are more likely to experience this gap, which puts them at a high risk of loneliness.

Previous research also presumed that different situations would affect individuals’ behavior through cognitive-affective units (such as encodings, expectancies, beliefs, affects, and goals; Mischel and Shoda, 1995; Yu and Yang, 2003). Life satisfaction is the subjective cognition about self, others, and surrounding environments, which was a negative predictor of loneliness (Nazzal et al., 2019), students who had trouble getting social support would feel dissatisfied with their lives, resulting in loneliness. If people hold positive beliefs about themselves and others, they will not experience excessive negative emotions. Negative emotions such as feelings of loneliness only arise when victims develop a cognitive bias and believe that he or she is unable to receive social support. Thus, we expect that school victimization affects Internet addiction through life satisfaction first and then through loneliness second (H4).

### The present study

In summary, the present study examines the mediating roles of life satisfaction and loneliness between school victimization and Internet addiction among Chinese adolescents. It has a few theoretical and practical implications, will complement the GST and provides some suggestions for future intervention studies on school bullying and Internet addiction. Based on the above discussions, we form the following four hypotheses:

*H1*: School victimization will correlate with Internet addiction positively.

*H2*: School victimization will associate with Internet addiction through loneliness.

*H3*: School victimization will associate with Internet addiction through life satisfaction.

*H4*: School victimization will associate with Internet addiction through the chain mediator of life satisfaction to loneliness.

## Materials and methods

### Participants and procedures

In this study, we used stratified cluster sampling to recruit participants. Participants were middle and high school students from a broader project focusing on the relationship between family environment and students’ mental health. In total, 3,363 adolescents (1,534 boys and 1,797 girls, 32 participants did not report their gender) participated in this study. They were recruited from 8 middle/high schools (110 classes), covering three urban and three rural districts of Beijing. The age range was 10.75–19.33 years old (*M* = 15.67; *SD* = 1.58). Participants were from 4 grades, including grade 7 (*N* = 605, *M_age_* = 13.59 years, *SD* = 0.47), grade 8 (*N* = 607, *M_age_* = 14.53 years, *SD* = 0.48), grade 10 (*N* = 1,033, *M_age_* = 16.56 years, *SD* = 0.45), grade 11 (*N* = 825, *M_age_* = 17.53 years, *SD* = 0.49), other 293 participants did not report their grade. Because of imminent graduation, this survey did not include students from grade 9 and grade 12. Among them, 22% of adolescents’ parents received education at secondary school or below, 37.5% received high school or vocational education, 30.9% have a college degree, and 9.6% have a master’s degree or above. Each student in the classroom completed self-reporting questionnaires after obtaining informed consent. It took approximately 20 min for a class to complete the set.

### Measures

#### School victimization

We used the Chinese version of the Olweus Bully/Victim Questionnaire (Dong and Lin, 2011) to measure school victimization. Thus, only the victimization subscale was used, which consists of seven items (e.g., being hit, kicked, pushed, or knocked intentionally by others). Participants were asked to report how frequently this behavior occurred over the past semester on a five-point scale (“0” = it has not happened to me, “1” = one time, “2” = two times, “3” = three or four times, “4” = five or more times). We used the total score of this subscale, and the higher score represents a higher level of school victimization. In the present study, Cronbach’s α for the subscale was 0.92.

#### Life satisfaction

We used a modified Chinese version of the Multidimensional Students’ Life Satisfaction Scale (MSLSS; Huebner, 1994; Tian and Liu, 2005) to assess life satisfaction. The MSLSS contains 25 items (e.g., “My family gets along well together”) that assess five important life domains of students (family, friends, school, living environment, and self), and each domain contains five items. This study examined the influence of school on adolescents’ Internet addiction, so we only contain three dimensions here (friends, school, and self). Participants rated each item on a 4-point Likert scale, ranging from 1 (totally disagree) to 4 (totally agree). We calculated the average score on each dimension and the total score, and the higher scores indicated higher satisfaction levels. Data from the present study showed good consistency for each dimension (for friends, α = 0.89; school, α = 0.87; self, α = 0.85).

#### Loneliness

Asher’s Child Loneliness Scale was used to measure each individual’s evaluation of his or her loneliness (Asher et al., 1984). The scale contains 20 items assessing four dimensions. Participants rated each item on a 4-point Likert scale ranging from very strongly disagree (1) to very strongly agree (4). The scale was translated into Chinese and tested by a previous study (Li et al., 2014; e.g., “It is easy for me to make new friends at school”). We calculated the average score on each dimension and the total score, with higher scores indicating higher levels of loneliness. The data exhibited good consistency (feeling of loneliness, α = 0.90; feeling of social adequacy versus inadequacy, α = 0.90; subjective estimation of peer status, α = 0.82; judgments about whether important relationship provisions are being met, α = 0.82; whole scale, α = 0.94).

#### Internet addiction

We used the Chinese version of the Internet Addiction Diagnostic Questionnaire (IADQ; Young, 1996; Wang et al., 2011) to measure adolescents’ Internet addictive behavior. It contains 10 items, and participants answered either “yes” (recorded as 1) or “no” (recorded as 0) to each item. The final score for Internet addictive behavior was computed by summing up one person’s points on all the items. A higher total score indicates a stronger tendency to conduct Internet addictive behavior. The Cronbach’s α in this study was 0.79.

#### Data analyses

SPSS 26.0 was utilized to conduct the correlations of all the key variables. Then, we employed the two-step procedure to analyze the mediation effects. First, we tested the measurement model to assess whether each latent variable was represented by its indicators. If the measurement model was accepted, then next, tested the structural equation model by using the MLR estimator of Mplus 8, which provided the standard errors and chi-square statistics for data with non-normal outcomes. Since the data were clustered within classrooms, the standard errors of parameter estimates and the chi-square test of model fit were computed using Mplus 8, taking the non-independence of observation into account. In addition, in Mplus maximum likelihood estimation, missing data due to attrition were allowed, but missing values were not imputed; instead, the method used all available information to estimate the model using full information maximum likelihood. Meanwhile, the item parcels were created for school victimization and Internet addiction to control inflated measurement errors by balancing the loadings and the average score of items used. Lastly, we used the model’s indirect command in Mplus 8 to test if the mediation effects were significant.

## Results

### Common method variance

We used Harman’s single-factor test to assess the common method variance. All items in this study were loaded into an exploratory factor analysis examining the unrotated factor solution using principal-component factor analysis with varimax rotation. Twelve components with initial eigenvalues greater than 1.0 were found *via* unrotated, principal-component factor analysis, and no dominating factor was found. The first factor accounted for 24.69% of the variances. This result demonstrated that there was only a small common method variance which can be ignored in this study.

### Measurement model

The measurement model consists of 4 latent constructs (school victimization, life satisfaction, loneliness, Internet addiction) and 13 observed variables. An initial test of the measurement model revealed a very satisfactory fit to the data: *χ^2^*/*df* = 13.43, *p* < 0.001; *RMSEA* = 0.06; *SRMR* = 0.05; *CFI* = 0.94; and *TLI* = 0.92. All the factor loadings of the indicators of the latent variables were reliable (*p* < 0.001), signifying that all the latent factors were well represented by their respective indicators. Means, standard deviations, and correlations for all measures can be seen in Table 1.

**Table 1:** Descriptive statistics and inter-correlations of the variables.

Variables	1	2	3	4
School victimization	1			
2. Life satisfaction	−0.17^***^	1		
3. Loneliness	0.21^***^	−0.61^***^	1	
4. Internet addiction	0.12^***^	−0.22^***^	0.22^***^	1
*M (SD)*	2.03 (0.97)	3.21 (0.49)	1.97 (0.72)	0.27 (0.25)

*p < 0.05; **p < 0.01; ***p < 0.001.

### Structural model

**Figure 1:**
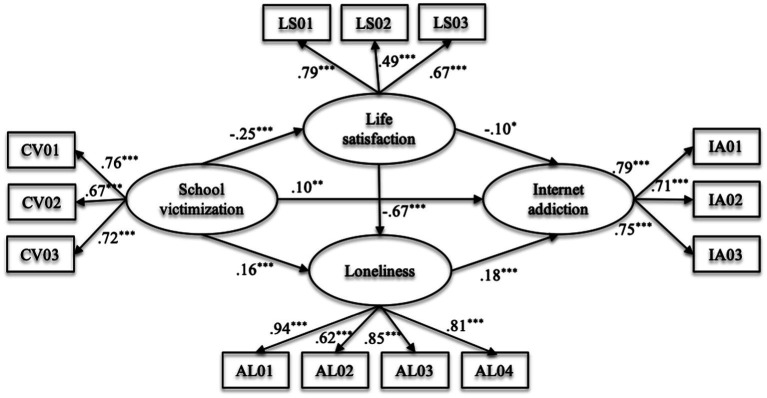
The structural model with gender and grade under control (*N* = 3,363). Note, the factor loadings were standardized. CV01-CV03, three parcels of victimization; LS01-LS03, satisfaction with friends, school, and self; AL1-AL4, four dimensions of loneliness; IA1-IA3, three parcels of Internet addiction. Form ^***^p < 0.001, ^*^p < 0.05.

Then, we conducted a structural model to explore the chain mediation effects of life satisfaction and loneliness. The model fitted our data well (*χ^2^*/*df* = 11.73, *p* < 0.001; *RMSEA* = 0.06; *SRMR* = 0.05; *CFI* = 0.93; and *TLI* = 0.91). The path from school victimization to Internet addiction was still significant (β = 0.10, *p* < 0.01), meaning that the link between two variables was only partially mediated by life satisfaction and loneliness. The significance of the mediating effects of life satisfaction and loneliness was tested in Mplus 8, which found that three mediation effect paths were significant in our study (Table 2). As in Figure 1, life satisfaction and loneliness mediated the relationship between school victimization and Internet addiction. Furthermore, the chained mediating path: school victimization → life satisfaction → loneliness → Internet addiction was also significant.

**Table 2:** Standardized indirect effect for the model.

Model pathways	Estimated	*p*
School victimization → life satisfaction → loneliness → Internet addiction	0.03	<0.001
School victimization → life satisfaction → Internet addiction	0.03	<0.001
School victimization → loneliness → Internet addiction	0.02	0.04

## Discussion

This study examined the association between school victimization and Internet addiction with the mediating effects of life satisfaction and loneliness among Chinese adolescents. The findings obtained from the present research were consistent with the literature findings, and our hypotheses were verified.

Results demonstrated a significant association between school victimization and Internet addiction. Adolescents with school victimization experience were more likely to have an Internet addiction. This finding was in agreement with other studies (Jia et al., 2018; Li et al., 2019). Depending upon the GST (Agnew, 1985), school is a fixed circumstance for adolescents. Experiencing victimization there can enhance unpleasant feelings and lead to tension for corrective action. When adolescents are victims at school, they may turn to the cyber world to reduce harm (Hsieh et al., 2016). The Internet is a haven for these adolescents to escape pressure from the real world. Additionally, they can also receive positive feedback from online friends. However, excessive use of the Internet may lead to Internet addiction.

The current study also tested the association among life satisfaction (including three dimensions: friend, school, and self), loneliness, and Internet addiction among adolescents. The results are shown in Table 1. Internet addiction was negatively correlated with life satisfaction and positively correlated with loneliness, implying that adolescents will score higher on Internet addiction if they possess lower levels of life satisfaction or feel lonelier. Prior studies support these correlational results (Çelik and Odacı, 2013; Akhter et al., 2020; Savolainen et al., 2020). Okur and Özekes (2020) confirmed that life satisfaction affected problematic Internet use negatively. Lonely individuals enjoy the online world because it allows them to relax when communicating with others online (Nowland et al., 2017). Thus, in some cases, loneliness results in Internet addiction if individuals obsessively indulge in the Internet and refuse offline interactions.

More importantly, the mediating effects of life satisfaction between school victimization and Internet addiction were demonstrated. Lower levels of life satisfaction would be observed in adolescents who have suffered from victimization as opposed to those who have not been bullied (Lázaro-Visa et al., 2019; Yang et al., 2021). Life satisfaction is cognition and evaluation of one’s life. Adolescents would have negative cognition and evaluation of themselves and others due to their victimization experience. To improve this matter, they are likely to expand their life to the Internet, even indulge in it, and thus get more satisfaction.

The model results also revealed that school victimization indirectly affected Internet addiction *via* loneliness. Adolescents who have been bullied are more likely to feel lonely than those without the experience of victimization (Newman et al., 2005; Estévez et al., 2009). Victims may feel isolated, uncared or no one understands them. Some victims may alienate their friends or classmates because they did not offer assistance or emotional support when victims were bullied. The Internet provided an alternative platform for victims to receive social support and emotional comfort. Moreover, the virtuality of the Internet can make victims avoid dealing with painful issues. Combined with the results, school victimization results in a decrease in life satisfaction and an increase in loneliness. Victims may turn to the online world, even engaging in Internet addiction behavior, which can not only escape real-world suffering but also compensate for the lack of life satisfaction and reduce loneliness.

Furthermore, the mechanism between life satisfaction and loneliness was established. Consistent with Lazarus’ cognitive theory of emotions, their loneliness level rises when adolescents are dissatisfied with their lives. The following factors contribute to a high level of loneliness: (1) dissatisfactory, low-quality, and meaningless social relationships, and individual needs cannot be satisfied; (2) irrational and false thinking; (3) deficit of social support. Given this, the chained mediating path exists. Adolescents experiencing school victimization had lower life satisfaction, felt more lonely, and finally contributed to Internet addiction. Adolescence is a critical and vulnerable period of psychological development during which adolescents’ developmental needs change, and their negative motivational and behavioral characteristics may increase. School is an important place for learning, entertainment, and social activity, which significantly impacts adolescents’ performance and behavior. School victimization is a negative change for adolescents, reflecting an imbalance between victims and their adverse environment. Victims’ intrinsic motivation and interest in school will dwindle in this situation, and withdrawal behavior will occur. In other words, an environment with school victimization fails to meet their psychological needs and brings out the negative cognitions and emotions of adolescence, eventually leading to problem behavior. Consistent with our hypotheses, the present study reveals that school victimization will decrease victims’ life satisfaction (including three dimensions: friend, school, and self) and increase their sense of loneliness. Then, Internet addiction emerges naturally. According to GST, school victimization is an environmental impediment for adolescents. It will destroy their trust in themselves and others, and this negative subjective cognition will evoke negative emotions, such as loneliness. Then, they will adopt retreating behavior, like plunging into the online world, to avoid this feeling of helplessness and negativity.

This study hypothesizes that school victimization affects Internet addiction through 4 pathways: (1) direct effect, (2) effect through loneliness, (3) effect through life satisfaction, (4) effect through life satisfaction first, and then through loneliness second. The study highlights the crucial roles of life satisfaction and loneliness in the link between school victimization and Internet addiction. It will broaden the theoretical understanding of GST. On a practical level, it can draw our attention to Internet addiction among individuals who experienced school victimization.

### Limitations

Of course, the present work has some limitations. First, only Beijing-based adolescent participants were used to conclude. Future studies should investigate whether our conclusions can be generalized to other developmental stages, geographical locations, and cultural contexts. Second, cross-sectional data cannot make causal inferences. Thus, future research should employ longitudinal or experimental approaches. Finally, this study focused on school bullying in the real world. However, cyberbullying is on the rise and may impact adolescent Internet addiction. More studies should be done to determine whether cyber victimization can produce the same results.

### Implications for practice, application, and theory

Despite these limitations, the current study has important theoretical and practical implications. Both school victimization and Internet addiction are severe and widespread adolescent issues, intervention often pays attention to one of the two aspects. Therefore, the exploration of the association and psychological mechanism between these two variables in the present study will provide some suggestions about adolescent development. First, GST highlights the emotional and behavioral responses to environmental strain. However, the present adds the consideration of the cognitive factor (life satisfaction) between environmental stress (school victimization) and emotional and behavioral response (loneliness and Internet addiction), which will broaden the understanding of GST. Second, as an environmental factor, school victimization strongly predicts Internet addiction. Therefore, improving school order and discipline is essential to change the tense atmosphere. Third, it is challenging for parents and teachers to identify whether adolescents are victims of school bullying. However, this study provides both cognitive (life satisfaction) and emotional indicators (loneliness) to help them identify and take intervene. Lastly, life satisfaction and loneliness mediate the link between school victimization and Internet addiction, suggesting possible remedies. Companies from friends, family, and teachers may help alleviate some adverse impacts of school victimization on Internet addiction. School activities and counseling services can help increase students’ life satisfaction, which may decrease the risk of being Internet addicts.

## Conclusion

The present study indicated that school victimization, life dissatisfaction, and loneliness are risk factors for developing Internet addiction. Additionally, life satisfaction and loneliness partially mediated the link between school victimization and Internet addiction. Adolescents who suffer from school victimization score lower on life satisfaction, experience more loneliness, and are more prone to be addicted to the Internet. This finding expands the current literature about Internet addiction. It also reminds us how to intervene in bullied adolescents and prevent possible adverse consequences.

## Data availability statement

The raw data supporting the conclusions of this manuscript will be made available by the authors, without undue reservation, to any qualified researcher.

## Ethics statement

The studies involving human participants were reviewed and approved by Ethics Committee of the Faculty of Psychology, Beijing Normal University. Written informed consent to participate in this study was provided by the participants’ legal guardian/next of kin.

## Author contributions

XS contributed by conceptualizing the study, analyzing data, collecting data, cleaning data, and writing some portions of the manuscript. RW contributed by writing the manuscript. All authors contributed to the article and approved the submitted version.

## Funding

This study was supported by the major projects from the Ministry of Education of Humanities and Social Science for the key research (14JJD190003) and Henan Philosophy and Social Science Foundation (2021CJY056).

## Conflict of interest

The authors declare that the research was conducted in the absence of any commercial or financial relationships that could be construed as a potential conflict of interest.

The reviewers SJ and ZY declared a shared affiliation with the author XS to the handling editor at the time of the study.

## Publisher’s note

All claims expressed in this article are solely those of the authors and do not necessarily represent those of their affiliated organizations, or those of the publisher, the editors and the reviewers. Any product that may be evaluated in this article, or claim that may be made by its manufacturer, is not guaranteed or endorsed by the publisher.
